# Secondary Caries Adjacent to Bulk or Incrementally Filled Composites Placed after Selective Excavation In Vitro

**DOI:** 10.3390/ma14040939

**Published:** 2021-02-16

**Authors:** Haitham Askar, Allam Al-Abdi, Uwe Blunck, Gerd Göstemeyer, Sebastian Paris, Falk Schwendicke

**Affiliations:** 1 Department of Operative and Preventive Dentistry, Charité—Universitätsmedizin Berlin, Aßmannshauser Str. 4-6, 14197 Berlin, Germany; allam.al-abdi@charite.de (A.A.-A.); uwe.blunck@charite.de (U.B.); gerd.goestemeyer@charite.de (G.G.); sebastian.paris@charite.de (S.P.); 2 Department of Oral Diagnostics, Digital Health, Health Services Research, Charité—Universitätsmedizin Berlin, Aßmannshauser Str. 4-6, 14197 Berlin, Germany; falk.schwendicke@charite.de

**Keywords:** bulk fill composite, dental caries, resin restorations, selective excavation

## Abstract

Objectives: selective caries excavation (SE) is recommended for deep carious lesions. Bulk fill composites (BF) may be considered to restore SE-cavities. We compared the susceptibility for secondary caries adjacent to BF versus incrementally filled composites (IF) in SE and non-selectively excavated teeth (NS) in vitro. Methods: in 72 extracted human premolars, artificial caries lesions were induced on pulpo-axial walls of standardized cavities. The lesions were left (SE) or removed (NS), and teeth were restored using two BF, GrandioSO x-tra/Voco (BF-Gra) and SDR/Dentsply (BF-SDR), and an IF, GrandioSO/Voco (IF-Gra) (n = 12/group for SE and NS). After thermo-mechanical cycling (5–55 °C, 8 days), teeth were submitted to a continuous-culture *Lactobacillus rhamnosus* biofilm model with cyclic loading for 10 days. Mineral loss (ΔZ) of enamel surface lesions (ESL), dentin surface lesions (DSL), and dentin wall lesions (DWL) was analyzed using transversal microradiography. Results: ΔZ was the highest in DSL, followed by ESL, and it was significantly lower in DWL. There were no significant differences in ΔZ between groups in DSL, ESL, and DWL (*p* > 0.05). Regardless of lesion location, ΔZ did not differ between SE and NS (*p* > 0.05). Conclusions: BF and IF both showed low risks for DWL (i.e., true secondary caries) after SE in vitro, and surface lesion risk was also not significantly different between materials. SE did not increase secondary caries risk as compared with NS. Clinical Significance: the risk of secondary caries was low after selective excavation in this study, regardless of whether bulk or incrementally filled composites were used

## 1. Introduction 

For deep carious lesions, selective caries excavation (SE) has been recommended, with soft or leathery carious tissue being intentionally left close to the pulp, while, in the periphery, only hard or firm dentin remains after excavation. While SE has been convincingly shown to reduce the risk of pulp exposure and pulpal complications, there is ambiguity in both clinical and in vitro data as to the restorative effects of SE [1,2,3]. It has been argued that, during SE, the amount of remaining carious tissue should be limited, with soft dentin only remaining in areas where pulp exposure is otherwise likely because the bond strength of dental adhesives to carious dentin are lower than to sound dentin, and, as carious hard tissues, come with a detrimental elastic modulus and impaired physical support for the restoration against masticatory forces [4,5]. In such circumstances, SE has not been found to come with higher risk of secondary caries or fracture than conventional, non-selective removal.

SE is often associated with extended cavities. For such cavities, bulk fill (BF) composites offer advantages over incrementally placed filling materials (IF), mainly as they save time and reduce the technical complexity of the restorative procedure. Although, the clinical evidence supporting bulk fills is limited [6,7,8], which is why in vitro studies are a useful means to compare the different material classes under controlled environments. Such environments also allow for studying specific restorative failures, like secondary caries (also termed caries adjacent/next to restorations, CAR). CAR has been found to be controlled by a number of factors, including the filling material used, the caries risk of the patient, and the experience of the operator [9,10,11,12,13]. Moreover, the application of occlusal loading (mainly during mastication) facilitates CAR development, most likely as fluid exchanges along the tooth-restoration interface are enhanced [14,15,16]. 

In the present study, we aimed to compare a new bulk fill, GrandioSO x-tra/Voco (Cuxhaven, Germany) (BF-Gra) against an established gold standard bulk fill, SDR/Dentsply (Konstanz, Germany) (BF-SDR), and an incrementally placed filling material, GrandioSO/Voco (IF-Gra), for their risk of CAR in vitro. In order to simulate CAR, a validated modeling system allowing to concomitantly load the restoration and cariogenically challenge the tooth-restorative complex using biofilms was used. Our null hypothesis (H_0_) was that the restoration material does not significantly influence the mineral loss (ΔZ) of secondary caries lesions in vitro. Our second hypothesis was that the carious tissue removal strategy, i.e., SE versus non-selective removal, was not significantly associated with ΔZ.

## 2. Methods

### 2.1. Study Design

In this study, seventy-two standardized proximal cavities were prepared in extracted human premolars to compare the risk of secondary caries adjacent to different bulk filling materials in association with SE. Artificial caries lesions were induced on the pulpal wall of the prepared cavities. Three filling materials were used in this experiment to evaluate the effect of the different bulk fill materials on the risk of secondary caries. Two bulk fill composites (BF-Gra and BF-SDR) and an incrementally placed filling IF-Gra, with n = 24/group. Each group was equally divided into half of the specimens being selectively excavated (SE) prior to placing the filling and the other half being non-selectively excavated (NS). All of the specimens were submitted to a cariogenic biofilm-model with occlusal cyclic loading for 10 days. The mineral loss (ΔZ) of the secondary caries lesions was assessed using transversal microradiography (TMR), with a separate analysis of enamel surface lesions (ESL), dentin wall lesions (DWL), and dentin surface lesions (DSL).

### 2.2. Specimens Preparation and Thermocycling 

In this experiment, 72 extracted caries free human premolars were used under an ethically approved protocol (ethics committee of the Charité—Universitätsmedizin Berlin EA4/102/14). The occlusal surfaces of all teeth were cut and polished in order to obtain a flat dentin surface and the roots were shortened, leaving 5 mm below the cemento-enamel junction [17]. 

A standard cavity (4 mm × 4 mm × 4 mm) was prepared in one (master) tooth and then transferred to the other cavities using copy-milling (Celay, Mikrona, Spreitenbach, Switzerland). Chemically induced lesions were created in the pulpal wall via storing the samples in a demineralising solution (pH 5.3, 37 °C) containing 50 mM acetic acid, 3 mM CaCl_2_ × H_2_O, 3 mM KH_2_PO_2_ and 6 mM methyl-hydroxydiphosphonate for 21 days. To control the pH level, the solution was monitored daily and, if necessary, adjusted using HCl or 10 M KOH [18]. In half of the specimens (n = 36), NS was performed, while, in the other half, the induced caries lesions were not removed simulating SE [18]. Specimens of each group were then randomly [19] allocated to the three experimental groups (n = 12); BF-Gra, BF-SDR, and IF-Gra. The sample size was mainly guided by the maximal capacity of the experimental setup (see below), but it was also in line with previous studies [20]. 

After cavity preparation, a universal adhesive bonding (Futurabond U, Voco, Cuxhaven, Germany) was applied in self-etch manner to the cavity for 20 s using micro-brush, followed by a gentle air blow for 5 s and light-curing for 10 s at an intensity of 1400 mW/cm^2^ from a distance <1 mm (Valo, Ultradent, Salt Lake City, UT, USA). A transparent matrix (Transparent band w/t/l 8.5/0.05/120 mm, Frasaco, Tettnang, Germany) was used for contouring. BF-Gra and BF-SDR were applied in 4 mm increments and then light cured for 40 s, as described, while IF-Gra was placed incrementally (2 mm increments), with each increment being light cured for 40 s. The excess material was removed with a scaler and no finishing and polishing of the margins were required given the options to shape margins using the matrix used. Because we were not interested intany occlusal wear, etc., the flowable BF (SDR) was also not further covered with any other resin composite, as would be done clinically.

After water storage for 100 days (37 °C, 100% humidity), the specimens were subjected to thermocycling, with 10,000 thermal cycles (Charité–Universitätsmedizin Berlin, Berlin, Germany) of sequential water bathing in 5 °C and 55 °C, with dwell and transfer times of 30 s and 5 s, respectively.

### 2.3. Bacterial Culture

*Lactobacillus rhamnosus* (LR, DSM 20021, DSZM, Braunschweig, Germany) was cultured for 48 h (37 °C, 5% CO_2_) on de-Man-Rogosa-Sharpe agar (MRS, Difco, Franklin Lakes, NJ, USA). The cultures were inoculated into 2.5 l MRS medium with 1% glucose and grown for 24 h to a density of approximately 7 × 10^6^ CFU/mL. LR has been used in a wide range of previous studies using caries models [17].

### 2.4. Masticatory Biofilm Model

**Figure 1:**
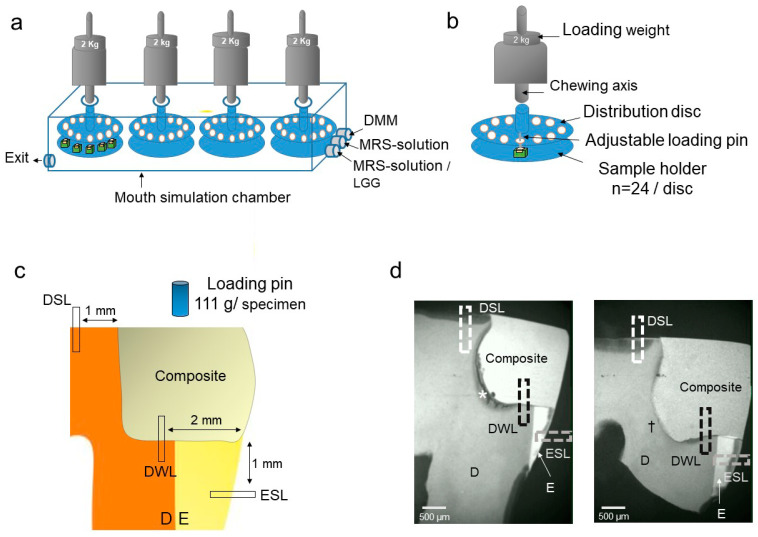
Experimental setup. (**a**) The masticatory biofilm model consisted for a chamber with three fluid entries and one exit as well as four loading axes and load distribution disc. (**b**) Loading of the distribution disc. Each disc could harbor 24 samples, with the loads being transferred from the chewing axes via the discs onto adjustable pins which loaded the samples. (**c**) Loading position on the specimen and measurement areas. D dentin, E enamel. (**d**) Microradiographs showing the mineral loss of different caries lesions. DSL, dentin surface lesion. DWL; dentin wall lesion. ESL, enamel surface lesion. * Selective excavation; residual lesion (SE). † Non-selective excavation (NS); removed lesion. D dentin, E enamel.

A custom-made masticatory biofilm model was used to simulate the mechanical and biological challenges of the oral cavity [15]. The model used four load distribution discs, with the specimens being loaded via adjustable stainless-steel pins (0.5 mm diameter), in order to transmit force onto each sample. The discs were fixed in a chamber within a dual-axis chewing simulation machine (Kausimulator CS-4.8, Willytech, Feldkirchen-Westerham, Germany) (Figure 1). The restored teeth were mounted to the four holding plates of the chewing simulator using cold-cure resin and the whole assembly (distribution disc and holding plate with the specimens) sterilized (121 °C, 2.1 bar, Tuttnauer 3870 ELV, Biomedis, Gießen, Germany) [17]. Afterwards, the holding plates were mounted within the artificial mouth chamber. Two kg loading weight were centrally applied vertically to each disc, transferring an occlusal load of 111 g per specimen. Loading was applied with a frequency of 0.16 Hz, 4 s loading, and 2 s of unloading. All of the specimens were subjected to a simulation period of 10 days at a loading frequency of 10 strokes per min. (equaling 144,000 loading cycles) [15].

After adjustment of the mechanical setup of the chewing simulator, LR biofilm was cultured at 100% humidity and 37 °C. The fluid flow within the chamber was managed using electric pumps (Seko PR1, Rieti, Italy). Three times daily (every eight hours), a simulated meal was provided, containing 1 L MRS plus 1% glucose solution, for 30 min. After an immersion time of 30 min. fluid was removed and a simulated salivary flow provided using 1 L sterile Modified Defined Mucin Medium (DMM) medium [21] for 30 min., followed by a saliva immersion time of 30 min., and removal of saliva. In the first of each daily cycle, we additionally provided LR overnight culture medium. 

### 2.5. Transversal Microradiography (TMR)

TMR was used to measure the mineral loss of the induced caries lesions. Note that TMR has been used as the gold standard for mineral loss measurement and to yield valid results, for example, as compared with micro-CT [22]. After the simulation period, specimens were removed and sectioned (Band Saw EXAKT 300cl, EXAKT Technologies, Norderstedt, Germany) mid-sagittally from mesial to distal. The sectioned surface was attached to an object holder, polished (silicon carbide papers 1200, 2500, 4000 grit), and thereby reduced to a thickness of 100 ± 10 μm (Band Saw EXAKT 400, EXAKT Technologies, Norderstedt, Germany). The polished sections were exposed to radiation (PW2213/20 tube, Panalytical, Kassel, Germany; PW 1730/10 generator, Philips, Eindhoven, The Netherlands) at 20 kV and 10 mA with an exposure time of 10 s. For calibration, a nine-step aluminum step wedge was introduced top the setup. Exposed films (Fine 71337, Fujifilm, Tokyo, Japan) were developed according to the manufacturer’s instructions under standardized conditions and digitally analyzed while using a transmitted light microscope and a CCD-video camera (XC 77 CE, Sony, Tokyo, Japan). The mineral content of the sound tooth structure in each specimen was calibrated to the step wedge gray scales, and then the mineral loss ΔZ (vol% × μm) measured using TMR software for Windows version 2.0.27.2 [23] (Inspector Research, Amsterdam, The Netherlands).

Mineral loss ΔZ (vol% × μm) was measured at three different areas: along the tooth-restoration interface to determine the DWL at 2 mm distance from the outer surface, along the outer surface of enamel in order to determine the ESL at 1 mm distance from the tooth-restoration interface and along the outer surface of occlusal dentin to determine DSL at 1 mm from the tooth-restoration interface (Figure 1). ΔZ of the artificially induced pulpal-wall lesions were also measured in order to check for any imbalances in that confounding variable between groups. 

### 2.6. Statistical Analysis 

Statistical analysis was performed using SPSS 22 [24] (IBM, Armonk, NY, USA). Normal distribution of the data was checked using the Shapiro–Wilk test. A comparison of ΔZ in different experimental groups (BF-Gra, BF-SDR and IF-Gra) was separately performed for ESL, DWL, and DSL using Generalized Linear Modelling (GLM) and Mann–Whitney–U-tests, respectively, with *p* ≤ 0.05 being considered to be statistically significant.

## 3. Result

**Figure 2:**
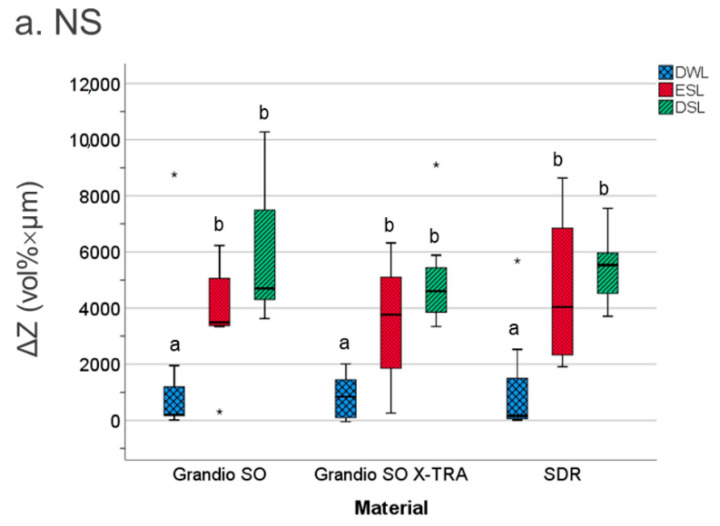

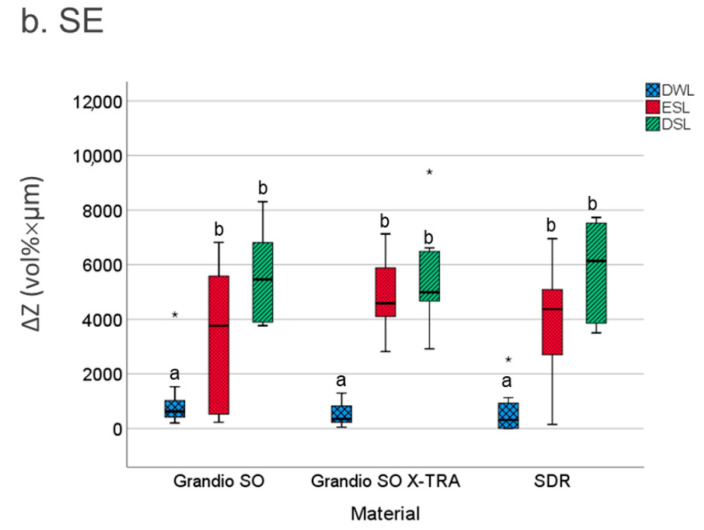
The mineral loss values ΔZ (vol% × µm) of caries lesions adjacent to the experimental filling materials at different positions. DWL: dentin wall lesion. SEL: enamel surface lesion. DSL: dentin surface lesion. Line: median; Box: 25–75th percentiles; whiskers: minimum–maximum, dots: outliers. Significant differences of mineral loss (ΔZ) between the different materials are indicated by different letters (**a**,**b**) (*p* < 0.05) (* outliers values).

ΔZ was the highest in DSL (median; 25/75th percentiles: 5221; 4111/6502 vol% × µm), followed by ESL (4160; 2572/5532 vol% × µm), and significantly lower in DWL (379; 102/1163 vol% × µm) (*p* < 0.01). For ESL, DWL, and DSL, neither the filling material nor the excavation strategy had a significant effect on the mineral loss ΔZ (*p* > 0.05, Mann–Whitney–U-test) (Figure 2). For artificially induced pulpal-wall lesions, no significant differences were observed in the ΔZ between the experimental groups (*p* > 0.05, Mann–Whitney–U-test).

## 4. Discussion

BF are an accepted and widely used restorative material, with a high profile research community characterizing and further enhancing this material class, which has received specific attention in the post-Minamata era as a possible amalgam replacement material [24]. While clinical data are emerging, pointing towards limited short-term differences between BF and IF [25], certain aspects, like secondary caries, are hard to assess clinically, as they require years-long follow-up periods beyond what is usually feasible in many controlled trials and what is useful when needing to make decisions towards employing new materials today or not [26]. Hence, in vitro studies are particularly useful for studying the risk of secondary caries adjacent to different restorative materials. A range of secondary caries models are available, each with specific strengths and weaknesses; in previous studies, a range of outcome measures have also been used for characterizing secondary caries [14,26,27,28]. In the present study, we employed a biologically grounded and previously validated model and mineral loss as an outcome parameter, as discussed further below. We investigated caries on various lesion locations, namely surface lesions in enamel and dentin (with a limited relationship to any kind of marginal interface, but possibly related to biofilm accumulation on the restorative material), and wall lesions along the interface between restoration material and tooth. We compared two different BF (one newly marketed and one established material) and an IF material serving as a reference in SE and NS cavities. The latter comparison was introduced as SE and NS have been claimed to possibly differ in their long-term restorative behavior, likely to the detriment of margin integrity and, thereby, secondary caries risk, which would be particularly relevant for BF, as these materials are a natural choice for deep and extended cavities. Overall, we refute the hypotheses of differences between materials or excavation strategies when it came to secondary caries and, hence, accept our null hypotheses; neither the restorative materials nor SE versus NS came with different mineral loss, regardless of the secondary lesions’ location.

Our findings require some more in-depth discussion. First, a range of previous in vitro studies as compared BF against each other and against IF for the restoration margin integrity or similar parameters. The majority of these studies did not find any significant difference in the margin integrity between BF and IF; in some cases, BF even showed higher margin integrity (this was especially the case when scalable BF, like BF-Gra, were used) [29,30,31]. Instead, the adhesive strategy has been confirmed as an important factor for margin integrity of all materials [30], in line with clinical data that were recently synthesized in a network meta-analysis [32], highlighting the relevance of the adhesive step for secondary caries risks. The same network meta-analysis also found BF and IF to yield similar risks of secondary caries, and generally confirmed the above said; secondary caries is a scarce event in controlled trials running over 1–3 years on average. Second, admittedly, comparisons of our findings against studies yielding margin integrity can only be made indirectly. Our outcome, mineral loss, suffers from limited granularity as compared with margin integrity, which is far more sensitive and may be able to detect even minor differences between the materials. Notably, margin integrity is only a surrogate, while secondary caries risk is tangible and relevant in the clinical arena. Future studies should aim to employ secondary caries and margin integrity analyses, while this was not readily possible given our attempt to (1) concomitantly perform loading and biofilm challenge and (2) assess mineral loss using microradiography, a destructive method, as discussed below. Third, we compared the secondary caries risk of restorations in SE and NS cavities. A range of studies on the biomechanical effects of intentionally sealed residual lesions are available. The first studies, leaving caries lesions on the pulpal floor of occlusal cavities in molars, found a devastating impact of these lesions on restoration fracture resistance [2]. In a range of follow-up investigations, mainly on proximally located lesions in premolars, did not confirm this impact when assessing margin integrity, cuspal deflection, the formation of enamel cracks, and fracture strength [3,33,34,35]. Thus, it was argued that the location of the residual lesion is relevant. In a recent finite element simulation, the relevance of lesion location was investigated once more and confirmed, with occlusal residual lesions showing highest stress along the filling-lesion interface, indicating failure risk. In contrast to what one would expect, smaller lesions induced higher stresses than larger ones, which was mainly due to large stress concentrations inside the filling walls [36]. In light of this, our study should be replicated with occlusal-pulpally located lesions, while for proximally located lesions (e.g., those that are frequently found in premolars), our findings corroborate previous studies and, hence, contribute to the growing body of evidence pointing towards no detrimental effect of SE on restorative integrity. 

This study has a number of strengths and limitations. First, we used an established experimental set-up, a validated simulation system, and an accepted outcome parameter. Notably, and as discussed, microradiography is a destructive and selective method for yielding mineral loss values, with only one thin section of each tooth being assessed (the assessment of two or more sections is technically challenging). In contrast, current micro-tomographic evaluations also allow highly dimensional assessment of mineral loss, and they provide a more holistic insight into lesion formation over time and in three dimensions. Second, the employed biofilm model is certainly a simplification, as only one—albeit relevant—strain was used. Notably, an undisturbed biofilm formation of LR has likely provided a highly cariogenic environment, which might not be found in the clinical situation. The demonstrated relevance of LR for dentin caries formation, however, supports using this key bacterium in models as ours, and possibly explains its frequent in in vitro ceries models [37,38]. Clinically, a wide consortium of species is related to secondary caries, while, for the purpose of this study, it is unclear whether using such a complex modeling system would yield additional insights and benefits. Notably, a further characterization of the biofilm itself (bacterial numbers, activity, architecture) might be undertaken to deepen our understanding of biofilms inducing secondary caries that are adjacent to different materials. Third, the employed approach for generating SE-lesions and NS-teeth is a simplification and only reflects partial clinical reality. Aspects, like pulpal reactions towards caries and possible reactionary dentin induction and remineralization processes are not at all reflected. The generated lesions have been found to resemble, mechanically, natural residual lesions, while admittedly bond strengths, etc., may differ [38]. Additionally, the removal of the induced carious dentin in the NS group was conducted by a single (calibrated) operator, i.e., is objective, and other operators might have left different degrees of dentin beneath the restoration. Given the agreement of our study findings with that of previous publications when it came to the impact of SE versus NS, though, we are confident that the impact of any such biases was limited. Fourth, we only compared three materials, all being placed using one bonding strategy. Clinically, a wider range of materials are available, and all of the employed restoratives can also be used in combination with other bonding strategies. A more comprehensive assessment may be warranted, given our scope of achieving mastication and biofilm challenge in a unique modeling setup did not allow for further specimens to be enrolled. However, given the consistent findings across all groups and the triangulation of our results with clinical and other in vitro studies, we remain confident that similar insights may emerge when using other established IF or BF materials. Finally, the specific application of the materials, without surface polishing or, for BF-SDR, any coverage with more wear-resistant materials was grounded into the needs and options in this study. Clinically, smooth contouring occlusally using a matrix is impossible and wear-resistance would be a relevant parameter to consider.

In conclusions, and within the discussed limitations, BF and IF both showed low risks for secondary caries, regardless of the location. In line with previous research, SE did not increase secondary caries risk as compared with NS when BF or IF were used. The risk of secondary caries was low after thermo-mechanical cycling, regardless of whether IF or Bf were used or SE or NS were performed.

## Data Availability

Not applicable.

## References

[1] Li T., Zhai X., Song F., Zhu H. (2018). Selective versus non-selective removal for dental caries: A systematic review and meta-analysis. Acta Odontol. Scand..

[2] Hevinga M.A., Opdam N., Truin G., Frencken J., Huysmans M. (2010). Does Incomplete Caries Removal Reduce Strength of Restored Teeth?. J. Dent. Res..

[3] Paris S., Schwendicke F., Seddig S., Müller W.-D., Dörfer C., Meyer-Lueckel H. (2013). Micro-hardness and mineral loss of enamel lesions after infiltration with various resins: Influence of infiltrant composition and application frequency in vitro. J. Dent..

[4] Innes N., Schwendicke F., Frencken J. (2018). An Agreed Terminology for Carious Tissue Removal. Oral Biofilms.

[5] Schwendicke F., Frencken J.E., Bjørndal L., Maltz M., Manton D.J., Ricketts D., Van Landuyt K., Banerjee A., Campus G., Doméjean S. (2016). Managing Carious Lesions: Consensus Recommendations on Carious Tissue Removal. Adv. Dent. Res..

[6] Veloso S.R.M., Lemos C.A.A., De Moraes S.L.D., Vasconcelos B.C.D.E., Pellizzer E.P., Monteiro G.Q.D.M. (2018). Clinical performance of bulk-fill and conventional resin composite restorations in posterior teeth: A systematic review and meta-analysis. Clin. Oral Investig..

[7] Schwendicke F., Göstemeyer G., Blunck U., Paris S., Hsu L.Y., Tu Y.K. (2016). Directly Placed Restorative Materials: Review and Network Meta-analysis. J. Dent. Res..

[8] Schwendicke F., Göstemeyer G., Stolpe M., Krois J. (2018). Amalgam Alternatives: Cost-Effectiveness and Value of Information Analysis. J. Dent. Res..

[9] Demarco F.F., Corrêa M.B., Cenci M.S., Moraes R.R., Opdam N.J. (2012). Longevity of posterior composite restorations: Not only a matter of materials. Dent. Mater..

[10] Dérand T., Birkhed D., Edwardsson S. (1991). Secondary caries related to various marginal gaps around amalgam restorations in vitro. Swed. Dent. J..

[11] Fontana M., Gonzalez-Cabezas C. (2000). Secondary caries and restoration replacement: An unresolved problem. Compend. Contin. Educ. Dent..

[12] Forss H., Widstrom E. (2004). Reasons for restorative therapy and the longevity of restorations in adults. Acta Odontol. Scand..

[13] Goldstein G.R. (2010). The Longevity of Direct and Indirect Posterior Restorations is Uncertain and may be Affected by a Number of Dentist-, Patient-, and Material-Related Factors. J. Evid. Based Dent. Pract..

[14] Khvostenko D., Salehi S., Naleway S.E., Hilton T.J., Ferracane J.L., Mitchell J.C., Kruzic J.J. (2015). Cyclic mechanical loading promotes bacterial penetration along composite restoration marginal gaps. Dent. Mater..

[15] Askar H., Brouwer F., Lehmensiek M., Paris S., Schwendicke F. (2017). The association between loading of restorations and secondary caries lesions is moderated by the restoration material elasticity. J. Dent..

[16] Kuper N.K., Opdam N.J.M., Bronkhorst E.M., Ruben J.L., Huysmans M.C.D.N.J.M. (2013). Hydrodynamic Flow through Loading and in vitro Secondary Caries Development. J. Dent. Res..

[17] Lehmensiek M., Askar H., Brouwer F., Blunck U., Paris S., Schwendicke F. (2018). Restoration integrity, but not material or cementation strategy determined secondary caries lesions next to indirect restorations in vitro. Dent. Mater..

[18] Schwendicke F., Eggers K., Meyer-Lueckel H., Dörfer C., Kovalev A., Gorb S., Paris S. (2015). In vitro Induction of Residual Caries Lesions in Dentin: Comparative Mineral Loss and Nano-Hardness Analysis. Caries Res..

[19] https://www.random.org/integer-sets/.

[20] Wong L., Sissons C. (2001). A comparison of human dental plaque microcosm biofilms grown in an undefined medium and a chemically defined artificial saliva. Arch. Oral Biol..

[21] Lo E.C., Zhi Q., Itthagarun A. (2010). Comparing two quantitative methods for studying remineralization of artificial caries. J. Dent..

[22] (2000). TMR 2000.

[23] (2009). SPSS 22.

[24] United Nations Environmental Programme (2013). Minamata Convention on Mercury.

[25] Arbildo-Vega H.I., Lapinska B., Panda S., Lamas-Lara C., Khan A.S., Lukomska-Szymanska M. (2020). Clinical Effectiveness of Bulk-Fill and Conventional Resin Composite Restorations: Systematic Review and Meta-Analysis. Polymers.

[26] Askar H., Krois J., Göstemeyer G., Bottenberg P., Zero D., Banerjee A., Schwendicke F. (2020). Secondary caries: What is it, and how it can be controlled, detected, and managed?. Clin. Oral Investig..

[27] Ferracane J.L. (2017). Models of Caries Formation around Dental Composite Restorations. J. Dent. Res..

[28] Askar H., Tu Y.-K., Paris S., Yeh Y.-C., Schwendicke F. (2017). Risk of caries adjacent to different restoration materials: Systematic review of in situ studies. J. Dent..

[29] Duarte J.C.L., Costa A.R., Veríssimo C., Duarte R.W., Filho S.C., Spohr A.M., Borges G.A., Correr-Sobrinho L. (2020). Interfacial Stress and Bond Strength of Bulk-Fill or Conventional Composite Resins to Dentin in Class II Restorations. Braz. Dent. J..

[30] Kimyai S., Kimyai S., Rahbar M., Ebrahimi A. (2020). Comparison of marginal adaptation of Class II cavities restored with bulk-fill and conventional composite resins using different universal bonding agent application strategies. Dent. Res. J..

[31] Paganini A., Attin T., Tauböck T.T. (2020). Margin Integrity of Bulk-Fill Composite Restorations in Primary Teeth. Materials.

[32] Askar H., Krois J., Göstemeyer G., Schwendicke F. (2021). Secondary caries risk of different adhesive strategies and restorative materials in permanent teeth: Systematic review and network meta-analysis. J. Dent..

[33] Silva P.F.D., Oliveira L.R.S., Braga S.S.L., Signori C., Armstrong S.R., Soares C.J., Cenci M.S., Faria-E-Silva A.L. (2018). Effect of selective carious tissue removal on biomechanical behavior of class II bulk-fill dental composite restorations. Dent. Mater..

[34] Santana M.L.C., Paiva L.F.S., Carneiro V.S.M., Gomes A.S.L., Cenci M.S., Faria-E-Silva A.L. (2020). Fracture resistance of extensive bulk-fill composite restorations after selective caries removal. Braz. Oral Res..

[35] Schwendicke F., Kern M., Blunck U., Dorfer C., Drenck J., Paris S. (2014). Marginal integrity and secondary caries of selectively excavated teeth in vitro. J. Dent..

[36] Weimann D., Morgenthal A., Schwendicke F., Fleck C., Razi H. (2021). Substantial regional differences in the biomechanical behavior of molar treated with selective caries tissue removal technique: A finite element study. Dent. Mater..

[37] Göstemeyer G., Schulze F., Paris S., Schwendicke F. (2017). Arrest of Root Carious Lesions via Sodium Fluoride, Chlorhexidine and Silver Diamine Fluoride In Vitro. Materials.

[38] Göstemeyer G., Kohls A., Paris S., Schwendicke F. (2018). Root caries prevention via sodium fluoride, chlorhexidine and silver diamine fluoride in vitro. Odontology.

